# Quantitative Detection of Epstein-Barr Virus DNA in Cerebrospinal Fluid and Blood Samples of Patients with Relapsing-Remitting Multiple Sclerosis

**DOI:** 10.1371/journal.pone.0094497

**Published:** 2014-04-10

**Authors:** Clementina E. Cocuzza, Fabrizio Piazza, Rosario Musumeci, Davide Oggioni, Simona Andreoni, Margherita Gardinetti, Letizia Fusco, Maura Frigo, Paola Banfi, Maria R. Rottoli, Paolo Confalonieri, Monica Rezzonico, Maria T. Ferrò, Guido Cavaletti, The EBV-MS Italian Study Group is formed, in addition to the cited, by the following persons:, Barbara Frigeni, Nerina Mascoli, Marta Conti, Carlo Antozzi, Francesca Andreetta, Elena Ambrosoni, Elsa Mainardi

**Affiliations:** 1 Dipartimento di Chirurgia e Medicina Traslazionale, Università di Milano-Bicocca, Monza, Italy; 2 Clinica Neurologica, A.O. S. Gerardo, Monza, Italy; 3 Dipartimento di Neurologia, Ospedale di Circolo e Fondazione Macchi, Varese, Italy; 4 Centro Sclerosi Multipla, A.O. Ospedale Papa Giovanni XXIII, Bergamo, Italy; 5 U.O. Neurologia IV, Centro Sclerosi Multipla, Fondazione Istituto Neurologico “Carlo Besta”, Milano, Italy; 6 Dipartimento di Neurologia, A.O Sant'Anna, Como, Italy; 7 Centro Sclerosi Multipla, Dipartimento di Neurologia, A.O. Ospedale Maggiore, Crema, Italy; Innsbruck Medical University, Austria

## Abstract

The presence of Epstein-Barr Virus (EBV) DNA in cerebrospinal fluid (CSF) and peripheral blood (PB) samples collected from 55 patients with clinical and radiologically-active relapsing-remitting MS (RRMS) and 51 subjects with other neurological diseases was determined using standardized commercially available kits for viral nucleic acid extraction and quantitative EBV DNA detection. Both cell-free and cell-associated CSF and PB fractions were analyzed, to distinguish latent from lytic EBV infection. EBV DNA was detected in 5.5% and 18.2% of cell-free and cell-associated CSF fractions of patients with RRMS as compared to 7.8% and 7.8% of controls; plasma and peripheral blood mononuclear cells (PBMC) positivity rates were 7.3% and 47.3% versus 5.8% and 31.4%, respectively. No significant difference in median EBV viral loads of positive samples was found between RRMS and control patients in all tested samples. Absence of statistically significant differences in EBV positivity rates between RRMS and control patients, despite the use of highly sensitive standardized methods, points to the lack of association between EBV and MS disease activity.

## Introduction

Multiple sclerosis (MS) is the most frequent chronic inflammatory disease of the central nervous system (CNS). Although the pathological features of this chronic demyelinating disease are well established [1], [2], little is currently known about the complex mechanisms that lead to the inflammatory process associated with MS.

Chronic persistence of Epstein-Barr Virus (EBV) infected cells in the peripheral circulation and/or in the CNS, possibly associated with lytic viral reactivation, has received increasing attention during recent years as a potential cause of MS onset and progression [3], [4], [5], [6], [7], [8], [9].

Previous investigations focused mainly on determining the presence of EBV latently infected B lymphocytes within the brain tissue [10], [11], [12], [13] and/or direct viral presence in the CSF [14], [15], [16], [17]. However, results have been highly discordant, with some authors reporting the presence of EBV-infected cells in the vast majority of MS cases [10], [13], whilst others have failed to demonstrate the presence of EBV or showed positivity in only a minority of MS brain tissues or CSFs [11], [12], [16], [18]. A recent focused experts workshop on the detection of EBV in MS brain [19] highlighted the possibility that divergent results may be due, at least in part, to technical issues, including methodological approaches and differences in the sensitivity and specificity of the various, mostly “*in house*”, detection assays used. These conclusions are likely to apply also to CSF studies.

This study addressed the possibility that the presence of EBV could have been missed in some of the previous studies, due to the pre-analytical and analytical methodologies applied to sample processing and analysis [20]. Standardized commercial methods were used in this study to obtain both high-yield automated recovery of microbial nucleic acids from different clinical matrices [21], [22], [23] and accurate quantification of EBV *EBNA-1* gene, using a previously described Real-Time PCR assay [24]. Moreover, a larger sample volume of CSF (1 ml), as compared to most previous studies, was analyzed in order to increase the sensitivity of detection.

Furthermore, in order to establish whether the presence of the virus was associated to latently infected cells or to viral lytic reactivation, EBV DNA detection was performed on both cell-free and cell-associated CSF fractions obtained from relapsing-remitting MS (RRMS) patients at the onset of a clinical relapse and compared to controls, affected by non-inflammatory or other inflammatory neurological diseases. The presence of EBV DNA was also determined in both peripheral blood mononuclear cells (PBMC) and plasma, collected at the same time of CSF sampling, in order to investigate the possible role of EBV systemic infection in the pathogenesis of MS, associated to either lympho-monocytes acting as carriers of virus particles from the periphery to the CSF, or to the release of free virus following lytic infection.

## Materials and Methods

### Ethics Statement

The study protocol was reviewed and approved by the Ethics Committee of the A.O. Ospedale S. Gerardo, Monza (PI investigator site) and by the Ethics Committees of the Ospedale di Circolo e Fondazione Macchi, Varese, A.O. Ospedale Papa Giovanni XXIII, Bergamo, Fondazione Istituto Neurologico “Carlo Besta”, Milano, A.O Sant'Anna, Como, A.O. Ospedale Maggiore, Crema, all in Italy. Written informed consent was obtained from the patients before study enrollment. Only adult patients aged 18 or more were included in the study.

### Patients

A total of 106 subjects, 55 affected by relapsing-remitting MS (RRMS) naïve to treatment and 51 controls (40 with non-inflammatory neurological diseases or resulted at the end of the diagnostic workout not to be affected by any neurological disease (NIND] and 11 with other inflammatory neurological diseases [OIND]), were included in this study. Patients' recruitment was carried out by seven clinical centers with specific expertise in MS management. MS diagnosis was made for all 55 patients during the acute relapse occurring at the time of recruitment, according to the revised McDonalds criteria [24] based on clinical and anamnestic findings and supported by the routine diagnostic workout. Investigations included at least brain MRI and CSF examination (routine cell count, IgG index: [IgG CSF/IgG serum]×[serum albumin/CSF albumin] with qualitative oligoclonal bands (OCB) analysis of intrathecal Ig-synthesis). All the RRMS patients had CSF examination within 2 weeks from the clinical relapse and they had active brain and/or spinal MRI with gadolinium enhancement in T1-weighed images. CSF samples were also obtained for diagnostic purposes from a group of control patients who underwent clinical and instrumental testing for symptoms suggesting acute or chronic neurological disorders of possible inflammatory or non-inflammatory origin; the final diagnosis in these subjects was supported by the appropriate diagnostic investigations.

### Specimen Collection and Processing

CSF and peripheral blood samples were collected from all patients at the same time point. Lymphocyte count was determined for all CSF samples. CSF samples were checked for microscopical blood contamination and samples with red blood cells observed at the standard counting chamber examination were excluded from the analysis. All remaining specimens were further processed as follows.

A volume of 1.5 ml of CSF was centrifuged at 180 g × 10 min at room temperature (RT), to separate cell-free CSF (supernatant) from the cell-containing CSF pellet, within 1 h following lumbar puncture.

Four ml of peripheral blood were centrifuged at 1000 g × 20 min at RT to obtain 3 aliquots of plasma. PBMC were obtained from 10 ml of peripheral blood by adding 10 ml of sterile saline, aliquoting in two 15 ml Ficoll gradients and centrifuging at 490 g × 30 min at 18°C. PBMC rings were then collected and further centrifuged at 700 g × 10 min at 18°C to obtain two separate PBMC pellets. All samples were stored at −80°C until analyzed.

### DNA Extraction

DNA extraction was carried out using the automated platform NucliSENS easyMAG (BioMérieux, Firenze, Italy) validated with a universal extraction kit based on magnetic beads (NucliSENS Nucleic Acid Extraction Reagents for easyMAG, BioMérieux, Firenze, Italy). This automated system has been specifically developed and validated for microbial nucleic acid extraction from different clinical matrices [21], [23], [25], [26] and has been proven to have a higher efficiency in viral nucleic acid recovery and a reduced risk of sample contamination than other available methods [21], [23], [25], [26]. To further improve viral DNA recovery, the “*specific*” nucleic acid extraction protocol was used, in accordance to the recommendations of the automated system's manufacturer, characterized by both a higher final elution temperature (70°C) and the use of a higher concentration of silica beads.

The efficiency of the automated system “*specific*” protocol in EBV DNA recovery was preliminary evaluated in this study by adding 4 different dilutions of commercially available EBV plasmids (EBV Q-PCR Alert Standard kit, ELITech Group, Puteaux, France) to aliquots of a known EBV negative cell-free CSF sample as well as including PCR-grade bi-distilled water as negative control to determine the potential risk of sample contamination during the nucleic acid extraction process. Nucleic acid extraction was carried out on three separate occasions to determine the intra-run and inter-run variability.

An initial starting volume of 1 ml of cell-free CSF was used for nucleic acid isolation and the nucleic acid extract was eluted in a final volume of 60 microl of Extraction Buffer 3 (NucliSENS Nucleic Acid Extraction Reagents for easyMAG, BioMérieux, Firenze, Italy).

CSF pellets (cells obtained following centrifugation of 1.5 ml of CSF sample) and PBMC (cells present in one of the two aliquots obtained following Ficoll gradient separation of 10 ml of peripheral blood) were first resuspended in 500 microl of Lysis Buffer (NucliSENS Nucleic Acid Extraction Reagents for easyMAG, BioMérieux, Firenze, Italy) × 10 min at RT and subsequently extracted and eluted in 100 microl of Extraction Buffer 3. Finally, 300 microl of plasma samples were processed for nucleic acid extraction and eluted in 100 microl of Extraction Buffer 3.

A positive internal control was added to clinical samples prior to DNA extraction (CPE – DNA – Internal control, Elitech Group, Puteaux, France) in order to evaluate the absence of PCR inhibitors, as recommended by the manufacturer's protocol. All samples were processed according to strict laboratory procedures to prevent sample contamination.

### Real-Time PCR for the Quantitative Detection of EBNA-1 Gene

Quantitative amplification of EBV *EBNA*-1 gene was carried out by means of a validated and certified (CE marked) standardized commercial kit, EBV Q-PCR Alert (ELITech Group, Puteaux, France), based on *EBNA*-1 gene detection, common to both EBV-1 and EBV-2 viral population, and on the use of Real-Time PCR, with previously described primers and probe (MGB) [23]. This highly specific and sensitive detection method was used to test and quantify separately CSF and blood samples divided into their cell-free and cell-associated fractions from 55 RRMS patients and 51 controls for EBV DNA.

Each run included a negative control (5 microl of sterile bidistilled water). Standard commercial EBV calibrators, 10^5^, 10^4^, 10^3^, 10^2^ copies/microl (EBV Q-PCR Alert Standard kit, ELITech Group, Puteaux, France) were also included in each run to obtain the standard calibration curve and as positive controls, as recommended by the protocol of the commercial kit. A further dilution of known DNA standards, corresponding to 10^1^ copies/microl, was also included to determine the lower limit of sensitivity of the assay for the accurate quantitative detection of low titer of EBV DNA samples.

Real-Time PCR assays were performed using the ABI PRISM 7900HT Sequence Detection System (Life Technologies, Monza, Italy). All samples were tested in duplicate. Multiple negative controls were included in each run. Five microl of extracted DNA was added to the Master mix (Q-PCR Alert AmpliMASTER, ELITech Group, Puteaux, France) to obtain a final volume of 25 microl. The PCR cycle protocol consisted of 2 min at 50°C, 10 min at 95°C, and 45 two-step cycles of 15 s at 95°C and of 1 min at 60°C.

The limit of EBV detection was 1 copy/reaction, corresponding to a threshold of 12 copies/ml in CSF. In parallel, the host cellular component was quantified in CSF pellet and PBMC samples, using a previously described *in house* assay for *CCR5* gene determination [27]. The EBV viral load present in these cellular samples was normalized on the basis of *CCR5* gene quantification, reflecting the number of cells present in the sample, and expressed as copies/10^2^ cells and copies/10^6^ cells in the CSF cell-associated pellet and PBMC, respectively.

### Statistical Analysis

The statistics program SPSS 20 (IBM SPSS Italia, Bologna, Italy) was used to perform all statistical analyses. For asymmetrical data Mann Whitney U test was used to compare distributions between cases and control subjects. The Chi-square (A/D) test was used to compare categorical variables. A p-value<0.05 was taken as statistically significant.

## Results

Results of routine CSF examination were consistent with the final diagnosis of RRMS in each patient and all the samples were available for analysis. With specific reference to RRMS patients, the lymphocyte count range was <1–14/microl (median = 7), the IgG Link index range was 0.50–1.58 and all but 3 subjects showed positivity for OCB. OIND and NIND subjects CSF examination revealed a lymphocyte count range <1–33/microl (median = 19) and <1–28/microl (median = 16), a IgG Link index range 0.17–0.27 and 0.36–0.85, respectively, and 1 subject showed positivity for OCB in each group.

The efficiency and reproducibility of the pre-analytical and analytical stages of the study were demonstrated prior to sample processing. In particular, NucliSENS easyMAG automated system with the use of the “specific” protocol for microbial nucleic acid extraction showed a very high efficiency in viral DNA recovery when spiking negative cell-free CSF samples with EBV DNA concentrations ranging from 10^5^ to 10^2^ copies/μl (Table 1). The correlation coefficient (*R* ^2^) for the standard curves based on different extraction runs of these dilutions was 0.999. All negative samples were demonstrated to be negative for all 3 runs confirming the very low risk of contamination by the use of the automated system, as indicated by previous studies [22].

**Table 1:** Correlation between expected results and automated extraction method performed as quality control for EBV DNA load measurement.

Expected results	EBV DNA load	
Log copies	Log copies	Ct mean
2	1.94	30.63
3	2.90	27.48
4	3.96	24.00
5	4.97	20.69

The table lists Ct value and log copies/reaction using standard commercial EBV calibrators (EBV Q-PCR Alert Standard kit, ELITech Group, Puteaux, France). Negative cell-free CSF samples were spiked with EBV DNA concentrations ranging from 10^5^ to 10^2^ copies/microl.

Results of EBV viral loads, obtained from clinical samples found to be positive for the presence of EBV *EBNA-1* gene, are shown in Table 2.

**Table 2:** EBV *EBNA-1* gene prevalence and viral loads in CSF and blood samples of 55 RRMS and 51 controls (OIND + NIND) patients.

EBV		RRMS	OIND	NIND	RRMS versus OIND		RRMS versus NIND	
					P value	OR	P value	OR
**Plasma**	Prevalence (%)	4/55 (7.3%)	0/11 (0%)	3/40 (7.5%)	0.356	-	0.967	1
	Median * V.L (range) *(copies/ml)*	126.5 (98–163)	-	189 (163–288)				
**PBMC**	Prevalence (%)	26/55 (47.3%)	7/11 (63.6)	9/40 (22.5%)	0.322	0.5	0.013	3.1
	Median V.L (range) *(copies/10^6^ cells)*	1328 (386–17131)	1005 (115–4045)	1412 (40–88506)				
**CSF cell free**	Prevalence (%)	3/55 (5.5%)	0/11 (0%)	4/40 (10%)	0.428	-	0.402	0.5
	Median V.L (range) *(copies/ml)*	216 (36–264)	-	56.5 (14–86)				
**CSF cell ass**	Prevalence (%)	10/55 (18.2%)	0/11 (0%)	4/40 (10%)	0.125	-	0.267	2
	Median V.L (range) *(copies/10^2^ cells)*	3 (0.2–25)	-	3 (1–9)				

PBMC  =  peripheral blood mononuclear cells; CSF  =  cerebrospinal fluid; V.L.  =  viral load; RRMS relapsing-remitting multiple sclerosis; OIND  =  other inflammatory neurological diseases; NIND  =  non-inflammatory neurological diseases or normal findings; OR  =  odd ratio.

*Median refers to positive samples.

Among 55 patients with active RRMS, 13 (23.6%) CSF samples had quantifiable *EBNA-1* DNA (3 in cell-free and 10 in cell-associated specimens – Table 2 and Table S1) and 30 (54.5%) blood samples were positive for *EBNA-1* DNA (4 from plasma and 26 from PBMC) as shown in Table 2 and S1.

In the overall control population composed by OIND + NIND, 8 samples obtained from 7 out of 51 (13.7%) subjects had quantifiable *EBNA-1* DNA in CSF (4 in cell-free CSF and 4 in CSF pellet, with two positive samples coming from the same NIND patient) and 19 (37.3%) had positive blood samples (3 plasma and 16 PBMC samples from 17 patients), as shown in Table 2 and S1.

Among the OIND cohort positivity for EBV DNA was never detected in the CSF, while 7 blood samples were positive. Among NIND patients the prevalence of *EBNA-1* DNA positive samples was 8/40 (20.0 2%) in CSF (1 both in cell-free CSF and pellet, 3 in cell-free CSF, 3 in CSF pellet) and 9/40 (22.5%) in blood samples (2 both in plasma and PBMC, 7 in PBMC only). Two of them were simultaneously positive in CSF and blood samples. Differences in EBV DNA positivities among the three groups were not statistically significant except for those demonstrated in PBMC samples of RRMS and NIND (p value = 0.013, O.R. = 3.1, Table 2).

Higher viral loads were found in both cell-free and CSF pellets samples from active RRMS patients as compared to control subjects, with the highest number of viral copies being observed in cell-free CSF samples (Table 2). The quantifiable viral loads embraced a wide range of values among considered groups based on cell-free and cell-associated samples. Among RRMS patients the median viral load in cell-free CSF positive samples was 216 copies/ml and in plasma was 126.5 copies/ml in positive samples , while in the cell-associated CSF a median value in positive samples of only 3 copies of EBV DNA/10^2^ cells was detected.

The median number of viral copies found in both positive CSF samples (cell-free and cell-associated CSF) from patients with MS (216 copies/ml, range 36–264 copies/ml and 3 copies/10^2^ cells, range 0.2–25 copies/10^2^ cells, respectively) and in CSF samples from patients without neurological diseases (56.5 copies/ml, range 14–86 copies/ml and 3 copies/10^2^ cells, range 1–9 copies/10^2^ cells, respectively) was comparable. As regards to OIND group, in the same samples no EBV DNA was found to be present.

On the other hand, the median number of viral loads found in positive blood samples (plasma and PBMC) from patients with MS (126.5 copies/ml, range 98–163 copies/ml and 1328 copies/10^6^ cells, range 386–17131 copies/10^6^ cells, respectively) and from patients without neurological diseases (189 copies/ml, range 163–288 copies/ml and 1412 copies/10^6^ cells, range 40–88506 copies/10^6^ cells, respectively) was comparable. As regards to OIND group, in plasma sample, no presence of EBV DNA was reported, while in PBMC sample a median viral load in positive samples of 1005 copies/10^6^ cells (range 115–4045 copies/10^6^ cells) was detected.

## Discussion

Technical issues related to the sensitivity and specificity of the detection methods were partly held responsible for the lack of unequivocal proof on the role of EBV infection in MS [19]. Although the presence of EBV PCR DNA in CSF is an indirect measure of the events occurring in brain tissue, its detection is somewhat relevant to the controversy.

This study aimed at providing evidence for the presence of EBV DNA in CSF and peripheral blood samples of RRMS patients, as compared to those of patients with other neurological diseases, by means of a validated, highly standardized and reproducible methods for viral nucleic acid extraction and molecular quantitative detection. All samples were processed rapidly after collection and stored under appropriate conditions prior to sample analysis. In order to improve recovery of hypothetical small numbers of viral particles present in the CSF a larger volume of sample was analyzed as compared to most previous studies. CSF and peripheral blood samples were also further separated in cell-free and cell-associated components prior to EBV DNA detection, in order to distinguish between possible latent and lytic EBV infections.

Our results showed similar EBV DNA positivities in cell-free and cell-associated CSF from RRMS and NIND subjects, while OIND subjects were all negative. Generally, slightly higher positivity rates were obtained when compared to previous investigations carried out on CSF samples from MS cases and controls [16], [28]. These differences could be explained by the larger volume of CSF sample analyzed, the improved efficiency in viral nucleic acid recovery, as a result of the DNA extraction method used, and the higher sensitivity of the standardized EBV molecular detection assay used in our study, allowing detection down to 10 copies/reaction or 120 copies/ml. However, in spite of the improved viral detection and quantification methods, this study did not provide evidence for an association between EBV and RRMS.

PBMC and plasma EBV viral load were also determined in this study as a measure of viral lytic infection and in order to evaluate if peripheral viral activity corresponded to disease activity. As observed in the CSF, in the peripheral blood samples positive for *EBNA-1* gene, EBV viral load was not significantly different in RRMS patients as compared to OIND and NIND subjects. These results are in keeping to those obtained in previous studies [29], [30], [31], although higher EBV positivities in PBMC were observed by Lindsey *et al* [32] (81% in MS subjects as compared to 89% in healthy controls) based on the presence of BamHIW repeat sequence (about 10 copies per EBV genome). However, when the authors analyzed the same samples for the presence of the single copy *LMP2a* gene, EBV was quantifiable only in 11% and 10.8% of MS and healthy control subjects, respectively. Higher, but not statistically significant, PBMC EBV DNA loads in patients with RRMS or with clinically isolated syndrome compared to healthy controls were also reported [32].

Differences in technical issues, such as the choice of the nucleic acid extraction method, the viral sequences to be detected, some present in single or multiple copies within the viral genome, differences in the methodologies and/or sensitivities of the detection assays used as well as the way of expressing viral load (such as EBV copies/10^6^ cells or copies/μg of DNA), make it somewhat difficult to appropriately compare results obtained in previously published studies [33].

In conclusion, this study used appropriately validated methods for both the pre-analytical and analytical detection of EBV DNA in order to provide improved standardized viral detection from different clinical samples. In spite of this improved methodological aspects and considering the relatively low sample size, our study confirms previous results showing lack of any significant difference in CSF and peripheral blood EBV DNA positivities and viral load between RRMS patients and the control groups.

## Supporting Information

Table S1 **Viral loads of EBV in CSF and blood samples of 55 RRMS and 51 controls (OIND + NIND) patients.** The table showed patient positivity for EBV by means of *EBNA-1* gene quantification in at least one sample. Each experiment was repeated twice and values reported in table are representative of the average of the two experiments. Patients numbers did not correspond to the original patient code. For the two control groups (OIND and NIND), final diagnosis was reported.
(DOCX)
