# Correction: Quantitative Detection of Epstein-Barr Virus DNA in Cerebrospinal Fluid and Blood Samples of Patients with Relapsing-Remitting Multiple Sclerosis

**DOI:** 10.1371/journal.pone.0103890

**Published:** 2014-07-25

**Authors:** The PLOS ONE Staff

The third author, Rosario Musumeci, should be noted as having also contributed equally to this work and is not the corresponding author.

There is an error in the email address for the corresponding author, Guido Cavaletti. The correct email address is: guido.cavaletti@unimib.it.

There is an error in the Funding section. The correct funding information is as follows: The study has been supported by Institutional research funds of the University of Milano Bicocca. The funders had no role in study design, data collection and analysis, decision to publish, or preparation of the manuscript.
