# Supplementary material for: Quantitative Detection of Epstein-Barr Virus DNA in Cerebrospinal Fluid and Blood Samples of Patients with Relapsing-Remitting Multiple Sclerosis
Source: PLoS One. 2014 Apr 10;9(4):e94497. doi: 10.1371/journal.pone.0094497 (PMC3983161; doi:10.1371/journal.pone.0094497)
Supplement: Table S1 — Viral loads of EBV in CSF and blood samples of 55 RRMS and 51 controls (OIND + NIND) patients. The table showed patient positivity for EBV by means of EBNA-1 gene quantification in at least one sample. Each experiment was repeated twice and values reported in table are representative of the average of the two experiments. Patients numbers did not correspond to the original patient code. For the two control groups (OIND and NIND), final diagnosis was reported. (DOCX) [file pone.0094497.s001.docx]

**Table S1 – Viral loads of EBV in CSF and blood samples of 55 RRMS and 51 controls (OIND + NIND) patients.**

| **Viral load in Samples Positive for *EBNA-1* gene** | | | | |
| --- | --- | --- | --- | --- |
| **RRSM** | **Cell-free CSF**  **(copies/ml)** | **Cell-Associated CSF**  **(copies/10^2^ cells)** | **Plasma**  **(copies/ml)** | **PBMC**  **(copies/10^6^ cells)** |
| 1 | 216 | - | - | 3237 |
| 2 | 264 | - | - | 4550 |
| 3 | 36 | - | - | 386 |
| 4 | - | 12 | - | 11276 |
| 5 | - | 25 | - | 854 |
| 6 | - | 1 | - | 8511 |
| 7 | - | 3 | - | 4044 |
| 8 | - | 18 | - | 2659 |
| 9 | - | 1 | - | 762 |
| 10 | - | 0.2 | - | 1556 |
| 11 | - | 1 | - | 525 |
| 12 | - | 6 | - | - |
| 13 | - | 2 | 163 | 3166 |
| 14 | - | - | 98 | 17131 |
| 15 | - | - | 104 | 1056 |
| 16 | - | - | 149 | 834 |
| 17 | - | - | - | 4386 |
| 18 | - | - | - | 413 |
| 19 | - | - | - | 812 |
| 20 | - | - | - | 1988 |
| 21 | - | - | - | 536 |
| 22 | - | - | - | 2242 |
| 23 | - | - | - | 1905 |
| 24 | - | - | - | 785 |
| 25 | - | - | - | 1328 |
| 26 | - | - | - | 584 |
| 27 | - | - | - | 936 |

**Footnotes**: The table shows patient positivity for EBV in at least one sample. Patients numbers do not correspond to the original patient code.

| **OIND group Patients**  **Final diagnosis** | **Cell-free CSF (copies/ml)** | **Cell-Associated CSF (copies/10^2^cells)** | **Plasma**  **(copies/ml)** | **PBMC**  **(copies/10^6^ cells)** |
| --- | --- | --- | --- | --- |
| Chronic inflammatory polyradiculoneuropathy | - | - | - | 115 |
| Acute encephalomyelitis | - | - | - | 2548 |
| Acute transverse myelitis | - | - | - | 563 |
| Isolated retrobulbar optic neuritis | - | - | - | 4045 |
| Inflammatory cranial polyneuropathy | - | - | - | 835 |
| Isolated retrobulbar optic neuritis | - | - | - | 1005 |
| Acute encephalomyelitis | - | - | - | 1500 |
| **NIND group Patients**  **Final diagnosis** | **Cell-free CSF (copies/ml)** | **Cell-Associated CSF (copies/10^2^cells)** | **Plasma**  **(copies/ml)** | **PBMC (copies/10^6^ cells)** |
| Degenerative dementia | 86 | - | - | - |
| Ischemic stroke | 45 | - | - | - |
| Thrombocytopenic purpura | 68 | 4 | - | - |
| Axonal idiopathic neuropathy | - | 2 | - | - |
| Axonal idiopathic neuropathy | - | - | 189 | 88506 |
| Axonal idiopathic neuropathy | - | - | - | 40 |
| Axonal idiopathic neuropathy | - | - | - | 745 |
| No evidence of neurological disease | 14 | - | - | - |
| Normal pressure hydrocephalus | - | 9 | - | 16628 |
| Axonal idiopathic neuropathy | - | 1 | - | 1412 |
| Motor neuron disease | - | - | 163 | - |
| Degenerative dementia | - | - | 288 | 3901 |
| No evidence of neurological disease | - | - | - | 507 |
| No evidence of neurological disease | - | - | - | 831 |
| No evidence of neurological disease | - | - | - | 9022 |
